# Optogenetics in Mice Performing a Visual Discrimination Task: Measurement and Suppression of Retinal Activation and the Resulting Behavioral Artifact

**DOI:** 10.1371/journal.pone.0144760

**Published:** 2015-12-11

**Authors:** Bethanny Danskin, Daniel Denman, Matthew Valley, Douglas Ollerenshaw, Derric Williams, Peter Groblewski, Clay Reid, Shawn Olsen, Jack Waters

**Affiliations:** Allen Institute for Brain Science, 551 N 34th St, Seattle, WA, 98103, United States of America; Dalhousie University, CANADA

## Abstract

Optogenetic techniques are used widely to perturb and interrogate neural circuits in behaving animals, but illumination can have additional effects, such as the activation of endogenous opsins in the retina. We found that illumination, delivered deep into the brain via an optical fiber, evoked a behavioral artifact in mice performing a visually guided discrimination task. Compared with blue (473 nm) and yellow (589 nm) illumination, red (640 nm) illumination evoked a greater behavioral artifact and more activity in the retina, the latter measured with electrical recordings. In the mouse, the sensitivity of retinal opsins declines steeply with wavelength across the visible spectrum, but propagation of light through brain tissue increases with wavelength. Our results suggest that poor retinal sensitivity to red light was overcome by relatively robust propagation of red light through brain tissue and stronger illumination of the retina by red than by blue or yellow light. Light adaptation of the retina, via an external source of illumination, suppressed retinal activation and the behavioral artifact without otherwise impacting behavioral performance. In summary, long wavelength optogenetic stimuli are particularly prone to evoke behavioral artifacts via activation of retinal opsins in the mouse, but light adaptation of the retina can provide a simple and effective mitigation of the artifact.

## Introduction

Modified microbial opsins have proven valuable molecules for the perturbation and interrogation of neural circuits [1–3]. In addition to offering reversible and temporally precise modulation of neural activity, these optogenetic tools can provide exquisite specificity [4]. Stimulation of selected sub-populations of somata or axons has been performed across a wide array of brain areas and has yielded insights into the roles of defined neurons, projections and circuits in various behaviors [5–7].

Recent advances include long-wavelength opsins that are activated by red light [8–11]. When combined with opsins that are activated at shorter wavelengths [12,13], these long-wavelength opsins permit bidirectional control of individual neurons or simultaneous, independent control of different populations of neurons. One disadvantage of stimulation with long wavelengths may be reduced spatial specificity. Brain tissue scatters long-wavelength light less than shorter wavelengths. As a result, long wavelengths propagate further through brain tissue than do shorter wavelengths, with the attendant danger that light may reach and activate opsins in regions distal from the site of stimulation, possibly including the endogenous opsins in the retina. An optogenetic stimulus that activates the retina may present a confound in behavioral experiments [14,15].

Here we describe an experimental protocol in which red illumination, delivered deep into the brain via an optical fiber, evoked a behavioral artifact in mice performing a visually guided discrimination task. We identify the mechanistic origin of the artifact, measuring electrical activity from the retina evoked by red illumination. Finally, we demonstrate that light adaptation of the retina, via an approach that would be easy to implement in many behavioral tasks, can almost eliminate the artifact without otherwise impacting behavioral performance.

## Material and Methods

### Mice and surgical procedures

All experiments and procedures were performed in accordance with protocols approved by the Allen Institute Animal Care and Use Committee. Mice were maintained on a reverse 12-hour light-cycle (off at 9am, on at 9pm) and all experiments were performed during the dark cycle.

Experiments were performed on male and female C57 mice (wild-type mice) or on transgenic mice expressing channelrhodopsin-2 (ChR2) or archaearhodopsin (Arch) in cholinergic neurons. Transgenic mice were produced by crossing Chat-IRES-Cre mice (Jax 006410, B6;129S6-Chat^tm2(cre)Lowl^/J) with Ai32D (Jax 012569, 129S-*Gt(ROSA)26Sor* ^*tm32(CAG-COP4*^ * ^*H134R/EYFP)Hze*^/J) or Ai35D (Jax 012735, B6;129S-*Gt(ROSA)26Sor* ^*tm35*.*1(CAG-AOP3/GFP)Hze*^/J). Where transgenic mice were employed, the optogenetic illumination experiments used a wavelength that does not activate the relevant opsin: 473 nm illumination for Arch-expressing mice and 589 or 640 nm illumination for ChR2-expressing mice.

For head restraint, a custom-made titanium plate was attached to the skull with C&B Metabond (Parkell S380), under isoflurane anesthesia (1–2.5%, inhaled). A guide cannula (C300GS-5/SPC, Plastics One) was implanted in the left hemisphere using stereotaxic coordinates (from bregma): -2.0 mm lateral, -0.5 mm posterior, -4.0 mm ventral. Surgery was performed at postnatal day 40–60. Before recovery from surgery, topical antibiotic (bacitracin-neomycin-polymyxin ointment) was applied to wound margins to minimize risk of infection. For alleviation of pain, mice were given ketoprofen (2-5mg/kg sub-cutaneous) immediately before recovery from anesthesia and twice daily for two days after surgery. Mice recovered for 7–10 days before starting water scheduling. Once experiments were complete, mice were euthanized by cervical dislocation under anesthesia (2–4% isoflurane, inhaled) or, usually, by transcardial perfusion under anesthesia (2–4% isoflurane, inhaled).

### Behavioral training and testing

The start of water scheduling (1–1.5 ml per day) coincided with 5–7 days of habituation, during which mice were handled, allowed to explore the behavioral apparatus (unrestrained) for ≤10 minutes per day and conditioned to head restraint in the behavioral box for ≤10 minutes per day. After habituation mice began training. Behavioral training and testing was performed in a custom-made light- and sound-attenuating chamber during the dark-cycle. Training and testing occurred once per day, 5 days per week, with no training (1.5 ml water per day) 2 days per week. Mice were supplemented with additional water or high-calorie food, as necessary, to maintain >80% of initial body weight.

During behavioral sessions the mouse was head-restrained and allowed to run on a 16.5 cm diameter disk while visual objects were presented on an LED monitor (Asus PA248Q) centered 15 cm from the right eye. The medial edge of the monitor was positioned 30° from the midline and the temporal edge at 150°. Hence the center of the monitor was at 90° from the midline. The luminance of the monitor ranged from 0 (black) to 90 (white) cd/m^2^.

The visual object was a 20° diameter circle containing a stationary grating, 0.15 cycles per degree with a half-cosine mask, generated by the PsychoPy package. The behavioral session was conducted with custom-written software in Python. Movement of the object was yoked to running speed via the rate of rotation of the running disk (0.17 degrees visual space/degree rotation of the disk), and the mouse collected a reward by slowing its running to select the visual object. A region subtending 40° in the center of the monitor was designated as the reward window. In order to select the object, the mouse held the object in the window for a minimum of 0.75–1.2 seconds. The reward for successful target selection was 5–10 μL of water, with mice performing 150–1000 trials in a single session. A behavioral session continued until the mouse received its daily allotment of water or until 60 minutes had passed, whichever was less. Where the mouse failed to collect all its water during behavioral training or testing, supplementary water was provided to bring the final volume to 1–1.5 ml.

Mice were eliminated from further study if, after 6 weeks of training, they routinely failed to run on the disk or stop to select the object (30% behavioral attrition rate). After mice learned to select the rewarded object (3–6 weeks), an unrewarded object (a novel orientation of the grating) was introduced randomly at a 1:1 ratio with the rewarded object. Mice learned to discriminate between the two objects within several sessions. Once a mouse learned to discriminate two high-contrast objects (criterion: d’>1.0 in 60% of sessions) additional low-contrast (0%, 25%, 38%) objects were added, but 0% contrast objects were not rewarded.

### Analysis of behavioral performance

Data analysis was performed using custom routines written in Python. Psychometric functions were fit to a Weibull distribution:

$$y=\;\gamma +\left(1-\;\gamma -\;\lambda \right)*\left(1-e^{-{\left(x/\alpha \right)}^{\beta }}\right)$$

Where α is the midpoint of the curve; β is slope at the midpoint; γ and λ are the lower and upper limits, respectively. Error bars for single-session behavioral data denote the 95% confidence-interval [16] implemented in the Python SciPy package.

The discriminability index (d’) was calculated as

d’= z(stop probability for rewarded objects) − z(stop probability for unrewarded objects)

where z is the inverse of the cumulative Gaussian distribution.

### Optogenetic illumination

Optogenetic illumination was provided via diode-pumped solid-state (DPSS) lasers (Opto-Engine: MGL-III-589 yellow-orange laser, MRL-III-640 red laser, MBL-III-473 blue laser). Illumination was delivered with a 300 μm diameter optical fiber (ThorLabs FT300UMT), inserted into the guide cannula so that the tip of the fiber extended just beyond the end of the cannula. Illumination was provided on 50% of trials and began as the leading edge of the object first appeared on the left side of the monitor and lasted until the object completely exited the screen (1–5 seconds depending on running speed). The inter-trial interval (time between the start of consecutive trials) was 4.6 ± 1.4 seconds (42 behavioral sessions, 11 mice). Hence fiber illumination, which occurred on 1 of every 2 trials on average, occurred for approximately 3 in every 9 seconds.

### Electroretinogram (ERG) recording

ERG recordings were performed under anesthesia (1–2.5% isoflurane) with periorbital silver wire electrodes placed in contact with the cornea. Two reference electrodes were inserted beneath the skin near the eyes. Amplifier headstages were positioned ~4 cm from each eye. Voltage signals were acquired (model 1800, AM systems) and digitized at 25 kHz with the Ecube acquisition system (White Matter LLC, Mercer Island, WA). Optogenetic illumination was presented for 200 milliseconds, with ≥1 second between stimuli. When pooling results across mice, to correct for differences in recordings conditions we normalized ERG voltage amplitudes to those evoked by blue illumination through the fiber tip (20 mW, 200 ms) with the fiber tip positioned outside the brain, in front of the head.

## Results

**Fig 1:**
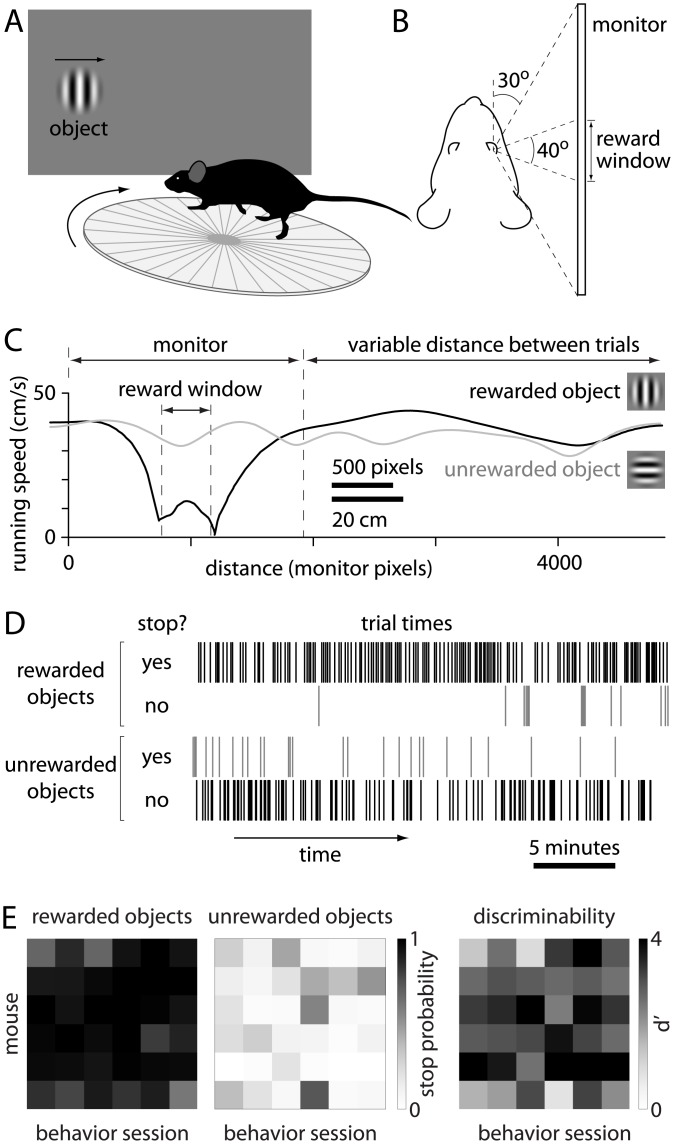
Performance of a visual discrimination task. (A) Schematic illustration of visual discrimination task. The mouse is head-restrained, on a running disk. Visual objects, displayed on a monitor, travel along the horizon from left to right. (B) Schematic illustration of the relative positions of mouse and monitor. (C) Running speed as a function of distance run by the mouse in two example trials. The position of the monitor, object and reward window are drawn to scale on the distance axis. (D) Summary of performance during a single example session, plot over time from the start to the end of the session (from left to right). Vertical lines indicate trials, sorted into four categories by object (rewarded vs unrewarded) and behavioral response (detected and undetected objects). The mouse collected rewards only on trials in which it indicated detection of a rewarded object (uppermost category). (E) Mouse-to-mouse and session-to-session variability in stop probability for rewarded and unrewarded objects and in discriminability (d'). Results are for 6 mice (rows) and across 6 sessions (columns).

Head-restrained mice were trained to perform a go/no-go visual discrimination task (Fig 1A). Visual objects were presented on a monitor in the right visual hemifield (Fig 1B). Objects moved horizontally across the monitor at a rate determined by the running speed of the mouse. A region in the center of the monitor was designated as the reward window and mice gained water rewards if they slowed their running speed sufficiently to hold the object in the reward window for a minimum of 0.75–1.2 seconds (optimized for each mouse).

Visual objects consisted of horizontally- or vertically-oriented sine wave gratings (diameter 20°, 0.15 cycles per degree on a 50% grey background), only one of which was rewarded. Mice learned to slow their running for rewarded objects and 'run past' objects that were not associated with a reward, with the run-speed trajectory diverging for rewarded and unrewarded targets (Fig 1C).

Mice performed one 0.5–1 hour session per day. Rewarded and unrewarded objects were interleaved randomly throughout a session, with mice observing 150–1000 objects per session. 69% (11 of 16) of mice learned the task to criterion performance, defined as discrimination between rewarded and unrewarded 100% contrast objects (d’>1) in >60% of sessions. Mice typically achieved criterion performance in 4–8 weeks. Mice that failed to reach criterion performance within 8 weeks were excluded from further study.

After training, performance was generally consistent throughout a session (Fig 1D) and was maintained for 3–6 months. Fig 1E summarizes performance for 6 mice across 6 sessions (18,578 objects), for which stop probability was 0.16 ± 0.03 (range 0–0.92) for 0% contrast objects; 0.17 ± 0.03 (range 0–0.73) for 100% contrast, unrewarded objects; 0.92 ± 0.02 (range 0.6–1) for 100% contrast, rewarded objects; and d' was 2.82 ± 0.17 (range 0.36–4.51) for 100% contrast objects.

**Fig 2:**
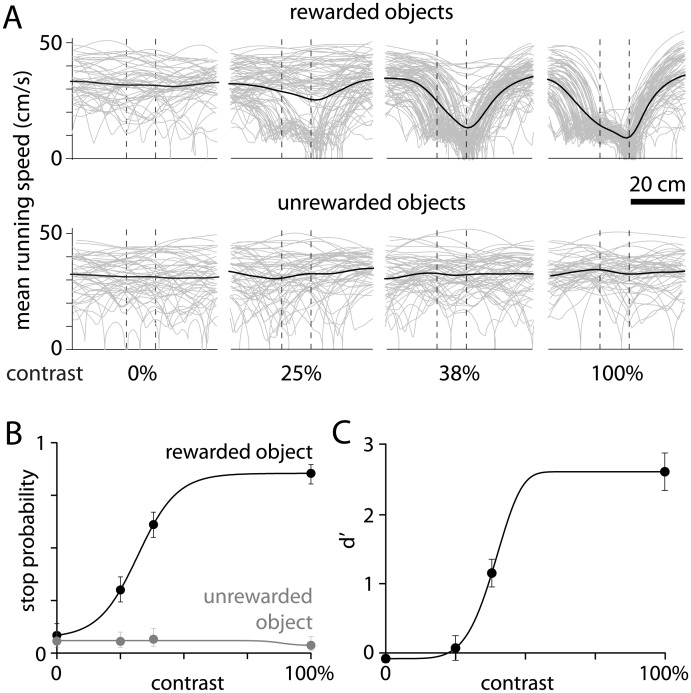
Performance across a range of contrasts. (A) Mean running speed trajectories, for a single session, for objects of contrasts from 0 to 100%, rewarded (upper row) and unrewarded (lower row) objects. Dashed vertical lines: limits of reward window. (B) Psychometric curves for rewarded (vertical) and unrewarded (horizontal) objects for the session illustrated in panel A. Line, fit to Weibull distribution; error bars, 95% confidence intervals. (C) Discriminability of rewarded and unrewarded objects as a function of contrast. Each point is the mean (± SEM) from 6 mice.

To more fully characterize performance across a range of difficulties, we presented objects at contrasts from zero to 100% (Fig 2). Stop probability was lower for 25% than for 100% contrast rewarded objects (0.16 and 0.92, respectively; p < 0.005, paired t-test), but did not change with contrast for unrewarded objects (0.16 and 0.17; p = 0.78, paired t-test). Mice had no biases for the two objects as they learned to perform the task equally whether trained to associate rewards with vertically- or horizontal-aligned objects (d’ 2.79 ± 0.52 and 2.69 ± 0.72, 5 and 2 mice, respectively).

### Illumination deep in the brain can impair performance

In optogenetics experiments, light is commonly delivered deep into the brain via a guide cannula and optic fiber. In behavioral experiments, and particularly during visual tasks, illumination could potentially be detected by the mouse and confound performance of the task.

**Fig 3:**
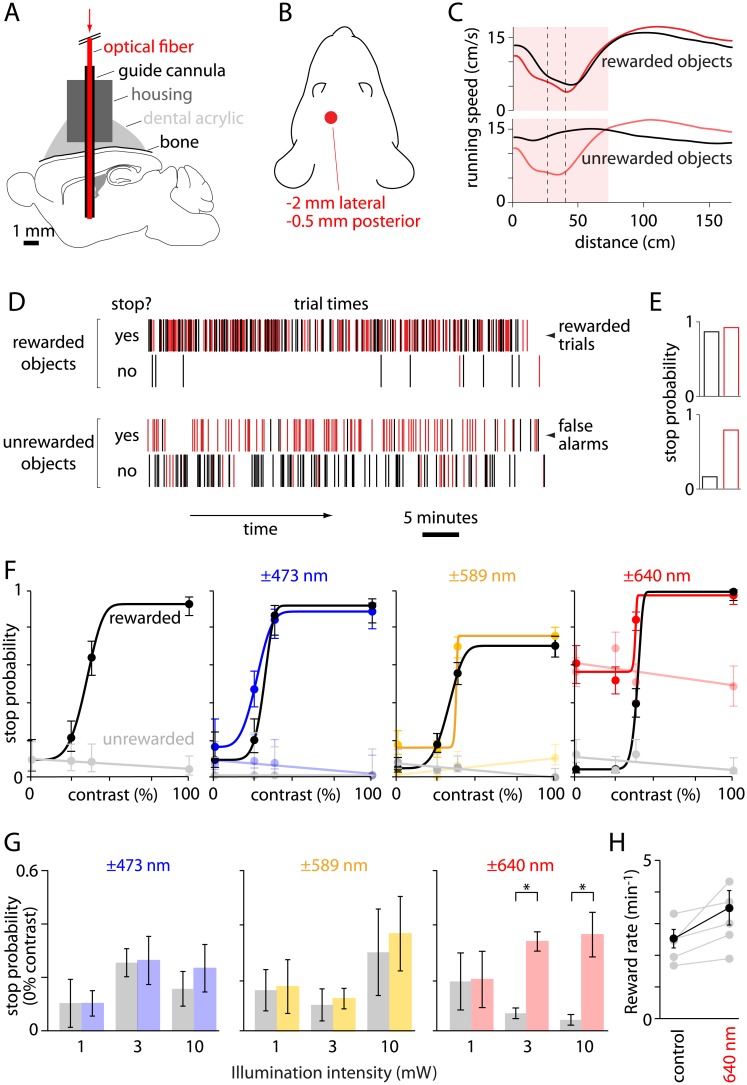
Behavioral artifact of deep brain illumination. (A) Schematic illustration of the implanted guide cannula, fiber and deep brain illumination. Drawn approximately to scale. (B) Schematic illustration of the fiber implant (red circle) viewed from the dorsal surface of the head, illustrating the position of the fiber relative to the eyes. (C) Mean running speed trajectory for a single session in which 50% of trials included 640 nm illumination. Rewarded and unrewarded objects were presented at 100% contrast with (red traces) and without (black traces) 640 nm illumination. (D) Summary of performance during the session illustrated in panel B. Trials with and without 640 nm illumination are illustrated with red and black vertical bars, respectively. (E) Stop probabilities for the session illustrated in panel C. (F) Psychometric curves for a single wild-type mouse across four sessions, with different deep brain illumination in each session: no deep brain illumination (left panel), 10 mW of 473 nm illumination (center left), 10 mW of 589 nm illumination (center right), 10 mW of 640 nm illumination (right). Each session included trials with (colored symbols and lines) and without (black, grey) illumination. Stop probabilities for rewarded and unrewarded trials are illustrated with darker and lighter colors, respectively. (G) Stop probabilities for zero-contrast objects (false alarm rates) for 1, 3 and 10 mW at 473 (blue), 589 (yellow) and 640 nm (red) illumination. Results for each intensity and wavelength were collected in a different session and compared to the stop probability without illumination in the same session (in black). Asterisks denote significant effects of illumination (p < 0.01). Numbers of mice: 6, 8 and 8 mice for 1, 3 and 10 mW of 473 nm illumination; 6, 5 and 6 mice for 1, 3 and 10 mW of 589 nm illumination; 4, 5 and 5 mice for 1, 3 and 10 mW of 640 nm illumination. (H) Reward rate under illuminated and control conditions, within the same session. Results from individual mice are illustrated in grey, mean ± SEM of 5 mice in black. Rewards summed across rewarded and unrewarded objects of all contrasts; numbers of trials of each contrast with and without illumination were approximately equal.

We implanted a cannula 4 mm below the pial surface of primary somatosensory cortex (Fig 3A) and delivered 473, 589 or 640 nm illumination throughout the transit of the object across the screen (typically ~3 seconds of illumination for a rewarded object and <1 second for an unrewarded object). Illumination was provided on 50% of trials in each session, in randomized order. 640 nm illumination exerted negligible effect on the animal's response to rewarded objects: stop probability to rewarded objects was unchanged (stop probability of 0.71 ± 0.11 without illumination, 0.79 ± 0.09 with illumination, 10 mice, p = 0.58, paired t-test), as was the latency of the response, measured from appearance of the object on the monitor to reward delivery (2.20 ± 0.04 s with 10 mW of 640 nm illumination, 2.03 ± 0.07 s without illumination, 5 mice, p = 0.06, paired t-test). However, 640 nm illumination provoked an increase in stop probability for unrewarded objects (increased 'false alarm rate', Fig 3C–3E). The increase in false alarm rate occurred only with relatively high intensities of long-wavelength illumination: no significant increase in false alarm rate was observed at 1 mW of 640 nm illumination or for 473 or 589 nm illumination up to 10 mW (Fig 3F and 3G).

The increase in false-alarm rate was not a non-specific response to illumination. When mice were presented with 640 nm illumination in the absence of a visual task, running speed was unchanged (for 10 mW, 640 nm illumination: 11.2 ± 1.6 cm/s with and 11.4 ± 1.6 without illumination, 6 mice, p = 0.63, paired t-test). Hence the increase in false alarm rate was not a reaction to illumination alone. Rather, mice appeared to be using 640 nm illumination as a cue to increase the rate of reward at low contrast. Consistent with this interpretation, during 10 mW, 640 nm illumination, reward probability increased. In the example session illustrated in Fig 3C–3E, reward probabilities in the absence and presence of 10 mW, 640 nm illumination were 0.04 and 0.51, respectively, for 25% contrast, rewarded objects. Furthermore, all mice gained more rewards on trials with than without 640 nm illumination (mean increase 38.6 ± 14.8%, 5 mice, Fig 3H). Hence mice were capable of using illumination as a cue that increased the probability of stopping on low contrast objects, thereby increased the rate at which they collected rewards.

### Activation of retina during deep brain illumination

**Fig 4:**
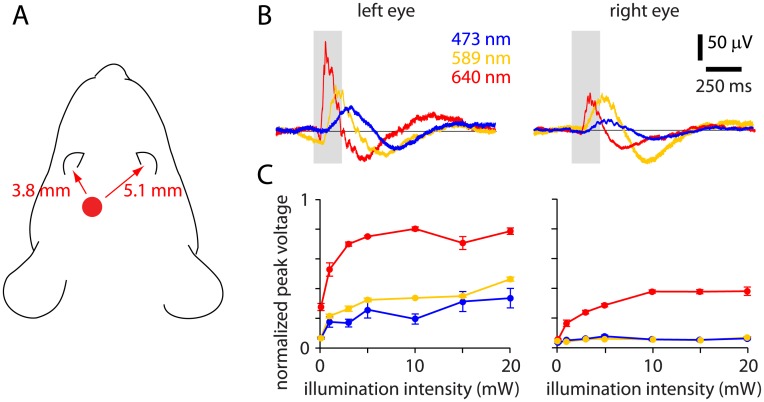
Deep brain illumination evokes activity in the left retina. (A) Example ERG recordings from left and right eyes of a mouse during 473, 589 and 640 nm illumination (with no illumination from the visual stimulus monitor). Duration of illumination (200 ms) is indicated in grey. Amplitudes are normalized to those evoked by illumination from an external light source (200 ms, 20 mW, 473 nm illumination via the fiber tip mounted in front of the eye). (B) Peak ERG voltage (normalized as in panel B) as a function of optogenetic illumination intensity at 473, 589 and 640 nm.

Our results suggest that mice can detect illumination delivered deep into the brain. To determine whether illumination was sufficient to evoke retinal activity in our preparation, we measured ensemble electrical activity in the retina during illumination via the implanted fiber. Using periorbital electrodes, we measured the electroretinogram (ERG) simultaneously from the left and right eyes of mice under isoflurane anesthesia. In the dark-adapted state, with the visual stimulus monitor off, illumination evoked retinal activity in both eyes (Fig 4A). Response amplitude was greater at the left than the right retina (Fig 4A and 4B), likely due to the relative proximity of the fiber tip to the eyes: in our experiments, the fiber tip was implanted ~3.8 mm from the left eye and ~5.1 mm from the right eye.

**Fig 5:**
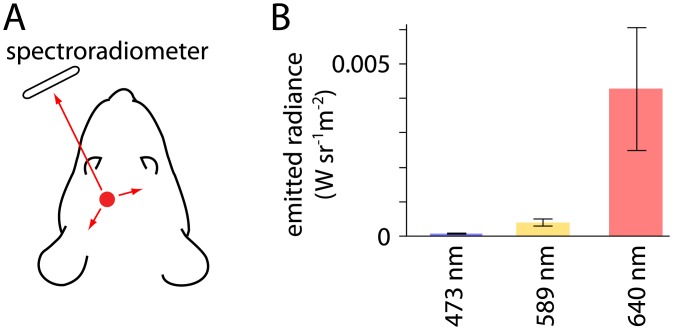
Measurement of light exiting the left eye. (A) Schematic illustrating the experimental arrangement during measurement of light emitted through the left eye. Light (red arrow) propagated from the implanted fiber (red circle) to a spectroradiometer, placed in front of the left eye. (B) Intensities measured by the spectroradiometer during 10 mW illumination through the implanted fiber at 473, 589 and 640 nm. 3 mice.

Response amplitude was greater during 640 nm than during 473 and 589 nm illumination, which may seem surprising since the M opsin, the principal visible wavelength-sensitive opsin in the mouse retina, is less sensitive to red than green or blue light [17–19]. However, propagation of light through brain tissue is wavelength-dependent, with stronger scattering at shorter wavelengths [5,15, 20, 21]. To gain an approximate measure of relative intensities at the retina during illumination through the implanted optical fiber, we measured light exiting the eye using a spectroradiometer positioned in front of the left eye (Fig 5). Illumination exiting the eye was ~50 times greater during 640 nm, 10 mW illumination than during 473 nm, 10 mW illumination (Fig 5B; radiance exiting the eye: 8.7 x 10^−5^ ± 2.0 x 10^−5^ W.sr^-1^.m^-2^ at 473 nm; 4.1 x 10^−4^ ± 1.0 x 10^−4^ W.sr^-1^.m^-2^ at 589 nm; 4.3 x 10^−3^ ± 1.7 x 10^−3^ W.sr^-1^.m^-2^ at 640 nm; 3 mice). Our results are consistent with propagation of light from the fiber tip, through the brain and onto the posterior aspect of the retina. The greater response at 640 nm likely indicates that red light from the fiber tip illuminates the retina at greater intensity than blue or yellow light and that this difference is greater than the wavelength-dependent difference in M opsin sensitivity.

**Fig 6:**
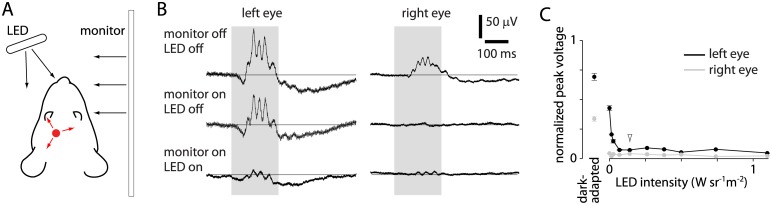
Light adaptation of left and right retinae. (A) Schematic illustrating the experimental arrangement of mouse head, visual stimulus monitor and LED, the latter placed to illuminate the left retina. (B) Example ERG recordings from left and right eyes of a mouse under different ambient illumination conditions: monitor and LED off (dark-adapted; top row); monitor on and LED off (middle row), and monitor and LED both on (lower row). Optogenetic stimulus was 200 ms, 10 mW, 640 nm illumination (grey). (C) Summary of the effects of LED illumination on the peak amplitude of the ERG voltage in different adaptation states. Left point (dark-adapted) with monitor and LED off. Remaining points were acquired with the monitor on and the LED providing differing illumination intensities. Points represent mean ± SEM (3 mice). Arrowhead marks 0.14 Wsr^-1^m^-2^.

ERG responses were observed at illumination greater than ~1 mW (Fig 4B), intensities at which the increase in false alarm rate was observed in mice performing the visual discrimination task. However, during visual discrimination behavior, with a monitor in the right hemifield (Fig 1B), the right retina, and perhaps the left retina, is unlikely to be dark adapted. We therefore made ERG measurements under conditions that more closely mimic our visual discrimination experiments (Fig 6A). In the presence of a monitor (at 50% grey luminance, 50 cd/m^2^) the amplitude of the ERG response from the right retina was almost eliminated, but there was little effect of the monitor on the response at the left retina (Fig 6B, 10 mW 640 nm illumination through the implanted optical fiber). With ERG we measured voltage responses to 200 ms illumination through the optical fiber. During behavioral experiments, fiber illumination typically lasted for several seconds. Nonetheless, our ERG measurements suggest that during visual discrimination mice detect 640 nm illumination via the left retina and that evoked activity in the left retina is necessary for the increase in false alarm rate.

We reasoned that light adapting the left retina might eliminate the increase in false alarm rate. To determine the amount of light needed to adapt the left retina, we illuminated the left eye with a white LED, placed ~8 cm in front of the left eye (Fig 6A). Left eye light adaptation almost eliminated the ERG response from the left retina at illumination intensities ≥0.14 Wsr^-1^m^-2^ (Fig 6B and 6C).

### Elimination of behavioral artifact

Finally, we tested whether light adaptation of the left retina eliminated the illumination-evoked increase in false alarm rate without otherwise impacting behavioral performance. The latter was of concern since the adapting LED illuminated primarily the left eye, but also likely illuminated the right retina, albeit at a lower intensity. In addition, the LED might have other undesirable effects such as adding a reflection from the surface of the monitor, which might reduce the effective contrast of visual objects. Hence the adapting LED might increase the difficulty of the task. For each mouse, we compared behavioral performance across sessions, with or without left retina adaptation. To minimize interference of the adapting LED in behavioral performance, we adapted the left retina with 0.14 Wsr^-1^m^-2^ (white LED) illumination throughout the session, which corresponds to the minimum intensity that almost eliminated retinal activation by 640 nm light.

**Fig 7:**
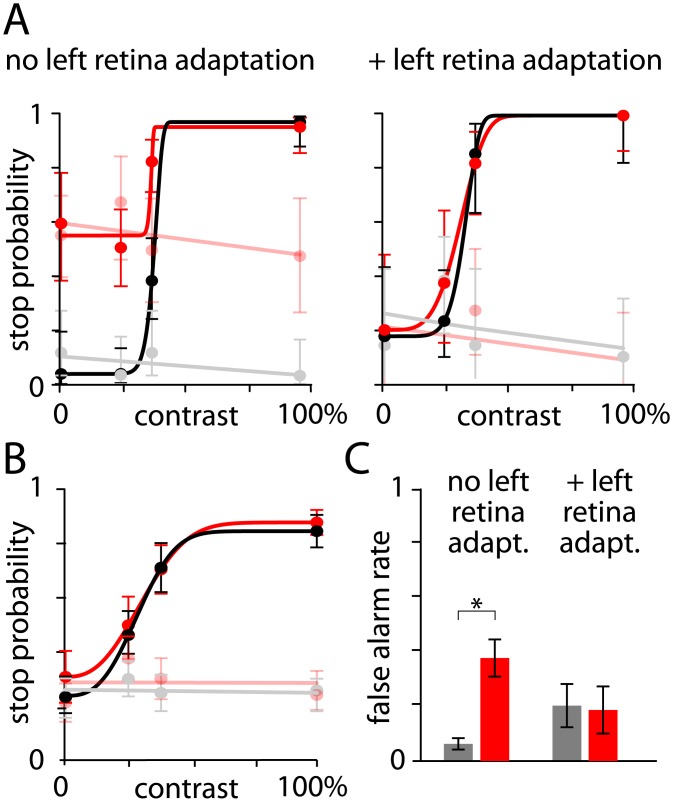
Illumination of the left eye eliminates the behavioral artifact. (A) Example of psychometric curves generated from single sessions without (left) and with (right) left retina adaptation (LED at 0.14 Wsr^-1^m^-2^). (B) Mean (± SEM) results and psychometric curves for 5 mice during left eye adaptation. (C) Summary of the change in false alarm rates with 10 mW, 640 nm illumination with and without left eye adaptation. 5 mice. Asterisk indicates p < 0.05, paired t-test.

In sessions with no left eye adaptation, false alarm rate increased during 10 mW, 640 nm illumination (Fig 7A; 5 mice, p < 0.05, paired t-test). In sessions with left eye adaptation, 10 mW, 640 nm illumination failed to evoke a change in false alarm rate (Fig 7A and 7B; 5 mice, p = 0.88, paired t-test). Furthermore, across the same sessions, there was no significant effect of left eye adaptation on false alarm rate in trials without 640 nm illumination (Fig 7B and 7C; 5 mice, p = 0.15, paired t-test) or on stop probability for rewarded objects (Fig 7B; for 100% contrast, rewarded targets 0.9 ± 0.05 with and 0.7 ± 0.1 without left eye adaptation, p = 0.16, paired t-test, 5 mice). Therefore, adaptation of the left retina with a constant white light source eliminated the behavioral artifact evoked by optogenetic illumination without impairing overall behavioral performance.

## Discussion

Our results indicate that long-wavelength illumination, 640 nm in our experiments, delivered deep into the brain via an optical fiber and at intensities commonly used in optogenetics experiments, can activate retinal photoreceptors and affect performance of a visual discrimination go/no-go task. In our experimental configuration, a mouse performed discrimination using its right eye and the fiber was implanted in its left hemisphere. In this configuration, behavioral performance was recovered by reducing the sensitivity of the left retina through light adaptation by an external source.

In our experiments, physical shielding blocked light exiting the fiber other than at the fiber tip. Hence retinal activation and the behavioral artifact were evoked by light exiting the tip of the fiber. Light from the fiber tip might have propagated though the brain, exited the mouse and entered the eye via the pupil. This route would likely result in equal activation of both retinae, but in our experiments the left retina was more strongly activated than the right, and the left eye was also closer to the fiber tip than the right eye. Hence it is likely that light from the fiber tip propagated rostrally through the brain and illuminated the retinae without exiting the mouse.

Propagation of light through brain tissue is wavelength-dependent, with stronger scattering at shorter wavelengths [5,15,20,21] and we estimated that illumination of the left retina was ~50-fold greater at 640 nm than at 473 nm. The wavelength sensitivity of the visible wavelength-sensitive M opsin in the mouse retina is maximal at approximately 500 nm and declines steeply at longer wavelengths. Sensitivity is reduced by an order of magnitude at ~570 nm and by 2 or more orders of magnitude at 640 nm [17–19]. Hence at equivalent intensities of illumination at the fiber tip, one might expect stronger activation of retinal photoreceptors at 473 nm than at 640 nm. Our results indicate the reverse: for equivalent intensities at the fiber tip, retinal activation and the resulting behavioral response is greater at 640 nm than at 473 nm. We conclude that, for longer wavelengths, the weaker scattering yields a sufficient illumination intensity at the retina to compensate for the reduced sensitivity, thus producing a visual response to 640 nm but not 473 or 589 nm nm light. Hence the intensity threshold that evokes significant retinal activation and a behavioral artifact is lower for red than for blue and yellow illumination. Our results indicate that the effective window of operation of optogenetics, between the minimum intensity required to evoke a photocurrent and maximum intensity that is imperceptible to the animal, is narrower for red- and for blue- and yellow opsins, underlining the need for red opsins which generate large photocurrents at relatively low illumination intensities.

Here we reported retinal activation upon illumination with ≥3 mW at the tip of an implanted fiber and we employed illumination of up to 30 mW (300 μm diameter fiber; calculated intensity per unit area of 42 and 424 mW/mm^2^). Many studies have reported successful activation of opsins, via implanted fibers *in vivo*, at lower illumination intensities than the maximum intensity we employed. However, intensities comparable to those employed here have been used in some studies, presumably because illumination intensity declines steeply with distance in brain tissue, thereby necessitating the use of high intensities at the fiber tip to activate opsins further from the implanted fiber (e.g. [5, 11, 22, 23]). There are multiple reasons to be cautious when using relatively high intensities, including the possibility of tissue damage, which likely occurs at ~100 mW/mm^2^ with blue light [24]. Our results indicate that one reason to be cautious is that high intensities can result in retinal activation and behavioral artifacts, particularly when using relatively long-wavelength, red illumination.

Clearly the success of our retinal adaptation strategy and the effect on behavioral performance depend on several factors, including the wavelength and intensity of the optogenetic illumination, the proximity of the fiber tip to the eyes and light adaptation due to illumination from the monitor. Whether the same strategy would be successful under different conditions, with a lower luminance monitor for example, would require further testing, but our results indicate that adaptation can be a successful strategy to eliminate visually mediated optogenetic artifacts under appropriate conditions.
