# Correction: Optogenetics in Mice Performing a Visual Discrimination Task: Measurement and Suppression of Retinal Activation and the Resulting Behavioral Artifact

**DOI:** 10.1371/journal.pone.0148883

**Published:** 2016-02-05

**Authors:** Bethanny Danskin, Daniel Denman, Matthew Valley, Douglas Ollerenshaw, Derric Williams, Peter Groblewski, Clay Reid, Shawn Olsen, Timothy Blanche, Jack Waters

Dr. Timothy Blanche is not included in the author byline. He should be listed as the ninth author and affiliated with the Allen Institute for Brain Science, 551 N 34th St, Seattle, WA, 98103, United States of America. The contributions of this author are as follows: Contributed reagents/materials/analysis tools, conceived and designed the experiments.
